# The intriguing phenomenon of cross-kingdom infections of plant and insect viruses to fungi: Can other animal viruses also cross-infect fungi?

**DOI:** 10.1371/journal.ppat.1011726

**Published:** 2023-10-26

**Authors:** Ida Bagus Andika, Xinran Cao, Hideki Kondo, Liying Sun

**Affiliations:** 1 College of Plant Health and Medicine, Qingdao Agricultural University, Qingdao, China; 2 Shandong Agricultural University, Tai’an, China; 3 Shouguang International Vegetable Sci-tech Fair Management Service Center, Shouguang, China; 4 Institute of Plant Science and Resources, Okayama University, Kurashiki, Japan; 5 State Key Laboratory of Crop Stress Biology for Arid Areas and College of Plant Protection, Northwest A&F University, Xianyang, China; Boston Children’s Hospital, UNITED STATES

## Abstract

Fungi are highly widespread and commonly colonize multicellular organisms that live in natural environments. Notably, studies on viruses infecting plant-associated fungi have revealed the interesting phenomenon of the cross-kingdom transmission of viruses and viroids from plants to fungi. This implies that fungi, in addition to absorbing water, nutrients, and other molecules from the host, can acquire intracellular parasites that reside in the host. These findings further suggest that fungi can serve as suitable alternative hosts for certain plant viruses and viroids. Given the frequent coinfection of fungi and viruses in humans/animals, the question of whether fungi can also acquire animal viruses and serve as their hosts is very intriguing. In fact, the transmission of viruses from insects to fungi has been observed. Furthermore, the common release of animal viruses into the extracellular space (viral shedding) could potentially facilitate their acquisition by fungi. Investigations of the cross-infection of animal viruses in fungi may provide new insights into the epidemiology of viral diseases in humans and animals.

The members of the fungal kingdom are highly diverse and ubiquitous. These eukaryotic microorganisms commonly exist as saprotrophs or associate with other living organisms through either parasitic/pathogenic or symbiotic/commensal relationships [1–3]. Therefore, most animals and plants in the natural environment, particularly those living in terrestrial ecosystems, are commonly colonized by fungi and fungus-like organisms such as oomycetes (kingdom Heterokonta) [4,5]. Depending on the type of relationship with the host, fungi interact with their hosts through intricate exchanges of various chemicals and biomolecular compounds [6,7]. Fungi absorb water and nutrients from the cells/tissues of the hosts while secreting molecules such as toxins, enzymes, effector proteins, lipids, and nucleic acids to facilitate or promote their infection. In response to fungal infection, the hosts secrete various compounds/molecules, including metabolites, peptides, proteins, and nucleic acids, that can be transferred into the fungal cells [8–13]. In addition, the host may absorb nutrients and other compounds from the fungi in a symbiotic relationship [14,15].

Like other cellular organisms, fungi and oomycetes are commonly infected with various viruses. Since the first finding of a mycovirus (fungal virus) in the early sixties, researchers worldwide have made great efforts to identify and characterize viruses from various taxa of fungi and oomycetes [16,17]. Thus far, most mycoviruses have been isolated from plant pathogenic fungi, primarily due to the objective of identifying mycoviruses that can reduce the growth and virulence of the fungal host; these may potentially be applied as biological control agents of crop fungal diseases [18,19]. Mycoviruses have also been identified from animal-associated fungi, including entomopathogenic and mammalian pathogenic fungi [20–27]. Further extensive identification of mycoviruses from human- and animal-infecting fungi and fungus-like organisms could extend our knowledge of the prevalence and diversity of mycoviruses in this fungal group and may lead to the discovery of mycoviruses with potential use as therapeutic antifungal agents [28,29].

Extensive studies on viruses infecting fungi have provided remarkable evidence of the events involved in natural virus transmission between plants and fungi. First, a number of mycoviruses have been found to have close taxonomic relationships or even high levels of genetic similarity with plant viruses [30,31]. The most striking examples are found in the genera *Alphapartitivirus* (family *Partitiviridae*) and *Alphaendornavirus* (family *Endornaviridae*), in which some mycoviruses are more similar to plant viruses than to other mycoviruses and vice versa [31,32]. In addition, in the family *Alphaflexiviridae*, some mycoviruses belonging to the genus *Botrexvirus* are similar to plant viruses belonging to the genus *Potexvirus* [33–35]. Previously, viroids (small plant parasites with a noncoding RNA genome) were found only in plants, but recently, viroid-like RNA agents have been discovered to naturally infect fungi [36,37]. Taken together, these observations may suggest the occurrence of past and relatively recent transmissions of viruses and viroids between plants and fungi. Second, fungal strains isolated from crop plants grown in the fields have been found to carry plant viruses. For instance, a strain of the phytopathogenic basidiomycete fungus *Rhizoctonia solani* isolated from a potato plant in the field was found to be infected with a plant virus, cucumber mosaic virus (family *Bromoviridae*) [38]. When a large number of filamentous fungal strains were isolated from various vegetable plants with viral diseases, around half of the fungal strains tested (169 strains) transiently carried diverse plant viruses (approximately 10 virus species) belonging to different genera and families [39]. A similar plant-to-fungus (also oomycete) transmission has also been observed for plant viroids. Among 117 filamentous fungal strains isolated from apple plants infected with apple scar skin viroid (family *Pospiviroidae*), 69.2% of the fungal strains carried this viroid [40]. Potato spindle tuber viroid (family *Pospiviroidae*) was detected in some natural isolates of *Phytophthora infestans* (an oomycete) [41]. Together, these observations suggest that during fungal colonization, fungi/oomycetes can acquire plant viruses and viroids that coinfect the plants.

Supporting the abovementioned view, bidirectional transmission of viruses and viroids between plants and fungi (also oomycetes) has been demonstrated using fungal inoculation experiments in plants under laboratory conditions [38,42–44]. These observations also suggest that fungi are suitable hosts for certain plant viruses and viroids. In fact, using artificial virus inoculation, various plant viruses and viroids have been shown to infect the yeast *Saccharomyces cerevisiae* (unicellular yeast fungus), oomycetes, and some filamentous fungi [38,42,43,45–52]. The mechanism of virus and viroid transfer across the boundary of plant and fungal cells remains obscure, although the extracellular secretory pathway responsible for the transport of proteins and nucleic acids from plants to fungi may be involved [30]. Notably, fungi have been shown to directly take up virus particles and RNAs that were extracellularly applied to the mycelia [42,49,53–55]. Further studies on the cross-infection of plant viruses in fungi could deepen our understanding of the spread, transmission, and evolution of plant viruses in nature.

Fungi are considered to be genetically more closely related to animals than to plants [56]. Some animal RNA and DNA viruses such as Flock House virus (family *Nodaviridae*), Nodamura virus (family *Nodaviridae*), influenza virus (family *Orthomyxoviridae*), human papillomavirus (family *Papillomaviridae*), bovine papillomavirus (family *Papillomaviridae*), and adeno-associated virus (family *Parvoviridae*) are shown to be able to replicate in yeast [57–62]. Since the coinfection of animal viruses and fungi can occur in the same host [63–68], it is worth questioning whether fungi can also acquire animal viruses during colonization. Notably, several animal viruses (mainly arthropod viruses) and fungal viruses show a close taxonomic relationship [69–81] (Fig 1). Moreover, in recent reports, several mycoviruses in the order *Bunyavirales* and the family *Rhabdovirdae* (order *Mononegavirales*) were identified to encode a putative precursor of glycoproteins or a glycoprotein similar to that encoded by animal-infecting viruses in the family *Phenuiviridae* or animal rhabdoviruses (subfamily *Alpharhabdovirinae*), respectively [75,77,82]. These suggest that the horizontal transfer of viruses between arthropods (and probably other animals) and fungi might have occurred in the past. A fungal single-stranded DNA virus, Sclerotinia sclerotiorum hypovirulence-associated DNA virus 1 (family *Genomoviridae*), isolated from the plant pathogenic ascomycete fungus *Sclerotinia sclerotiorum* was acquired by *Lycoriella ingenua* (Diptera: Sciaridae), a fungus-feeding insect, and replicated in the insect. Furthermore, viruliferous *L*. *ingenua* could transmit this virus to *S*. *sclerotiorum* [83]. Interestingly, entomopathogenic fungi (*Ascosphaera apis* and *Aspergillus tubingensis*) isolated from honey bees with the chalkbrood disease were found to be infected with various honey bee viruses belonging to the families *Dicistroviridae* and *Iflaviridae* [84,85]. These observations provide examples of virus transmission from an insect to a fungus.

**Fig 1:**
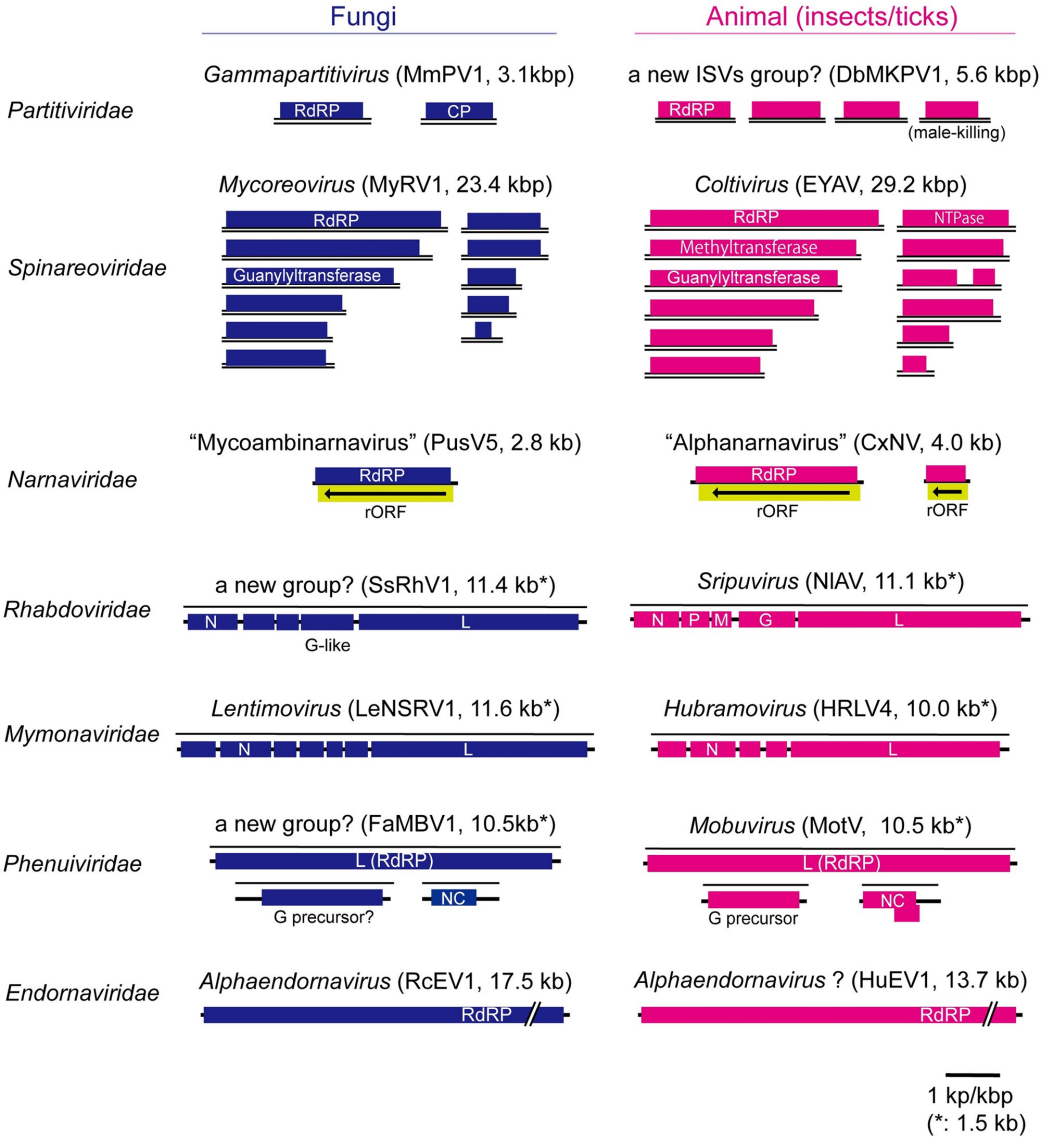
Schematic genome structures of representative animal viruses (arthropod viruses) and fungal viruses (phyto- and entomopathogenic fungal viruses) with close taxonomic relationships. dsRNA viruses, MmPV1: Metarhizium majus partitivirus 1 (virus accession numbers OL518956/OL518957), DbMKPV1: Drosophila biauraria male-killing partitivirus 1 (LC704637–LC704640), MyRV1: Mycoreovirus 1 (AY277888–AY277890, AB179636-AB179643), EYAV: Eyach virus (AF282467–F282478); ambisense RNA viruses, PusV5: Puccinia striiformis virus 5 (ON040903), CxNV1: Culex narnavirus 1 (MW226855/MW226856); negative-sense RNA viruses, SsRhV1: Sclerotinia sclerotiorum rhabdovirus 1 (MT706019), NlAV: Niakha virus (KC585008), LeNSRV1: Lentinula edodes negative-strand RNA virus 1 (LC466007), HRLV4: Hubei rhabdo-like virus 4 (KX884403), FaMBV1: Fusarium asiaticum mycobunyavirus 1 (MZ969068–MZ969070), MotV: Mothra virus (KX272883–X272885); positive-sense RNA viruses, RcEV1: Rhizoctonia cerealis endornavirus 1 (KF311065), HuEV1: Hubei endorna-like virus 1 (KX883776). “Mycoambinarnavirus” and “Alphanarnavirus” are proposed as genera within the family *Narnaviridae*. SsRhV1 and FaMBV1 encode glycoprotein-like (G-like) proteins similar to those found in classical rhabdoviruses (G protein) and animal phenuiviruses (Gn/Gc precursor protein) [97,98]. The G or Gn/Gc proteins have been shown to be involved in the virion formation and are likely to play a role in modulating the cell entry into arthropod vectors or other animal hosts. CP: coat protein, RdRP: RNA-dependent RNA polymerase, rORF: reverse open reading frame, N or NC: nucleocapsid protein, P: phosphoprotein, M: matrix protein, G: glycoprotein, ISV: insect-specific virus.

The transfer of molecules between biotrophic fungi and plants commonly occurs via passage through the cell membranes and extracellular interfaces such as the extrahaustorial matrix and apoplastic space [86–89]. On the other hand, some opportunistic invasive human fungal pathogens such as *Candida albicans*, *Cryptococcus neoformans*, and *Aspergillus fumigatus* are capable of penetrating the host cells through endocytosis and/or the lysis of cell membranes [90–92]. This infection characteristic of human/animal fungal pathogens may facilitate the acquisition of viruses by the fungi. Moreover, the release of virus progenies into the extracellular space (viral shedding) through cell lysis, budding, exocytosis, apoptosis, or extracellular vesicles at later stages of infection, a common characteristic of animal viruses [93,94], may provide an opportunity for the contact of animal viruses with fungi (Fig 2). Finally, the ability of fungi to directly take up macromolecules such as RNA and virions may allow for the nonspecific entry of viruses into the fungal cells (Fig 2); this process is distinct from the canonical cell-surface receptor-regulated entry of viruses into animal cells [95,96]. The possible roles of fungi and fungus-like organisms as alternative hosts or biological reservoirs for animal viruses warrant further exploration. As fungi could potentially play a role in the dissemination of animal/human viruses in the environment, preventive measures to control viral diseases should include sanitation programs targeting fungi and fungus-like organisms.

**Fig 2:**
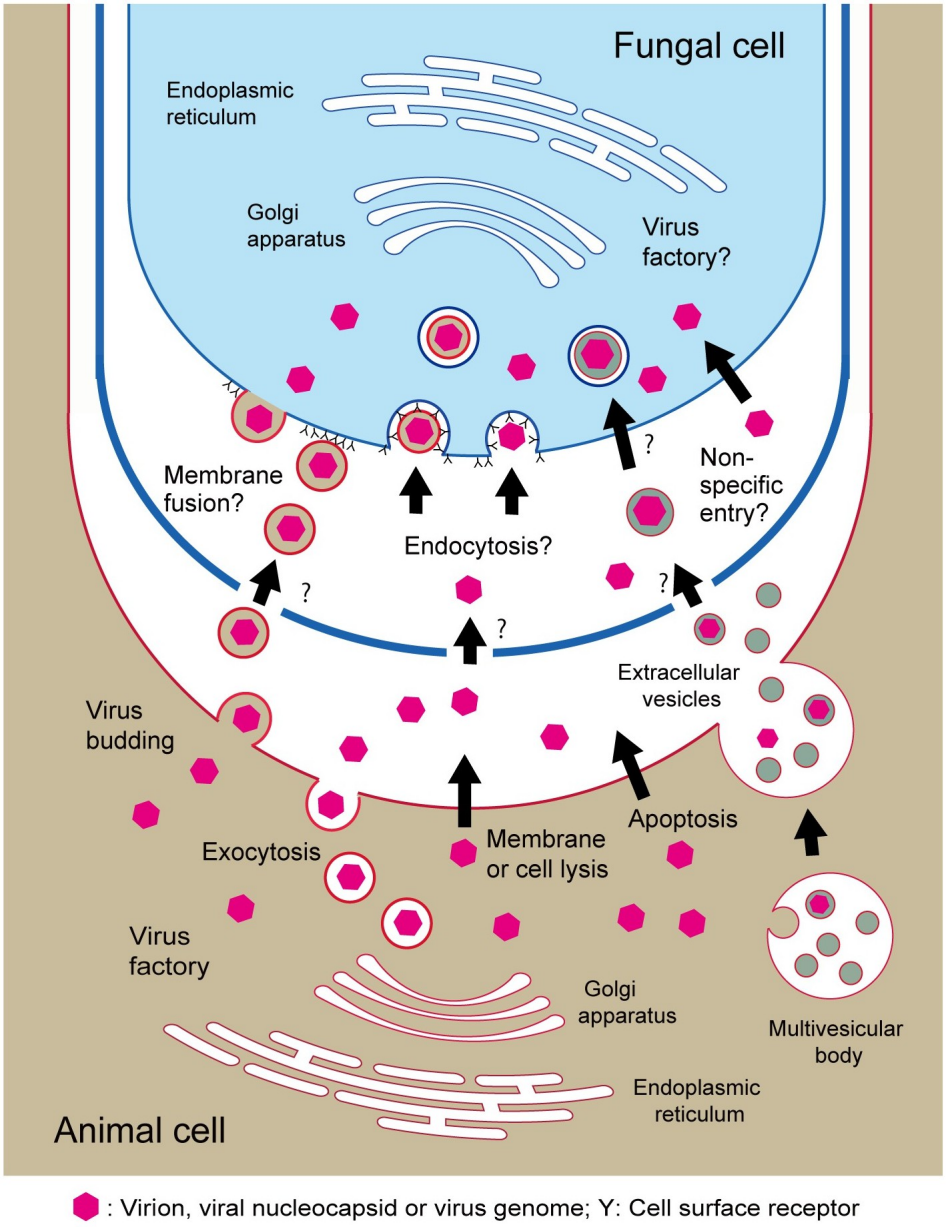
An illustration depicting the possible transfer of animal viruses from an animal cell to a fungal cell. At the later stages of infection, the progeny of animal viruses are commonly released into the extracellular space of animal cells (known as viral shedding) through various processes such as cell lysis, budding, exocytosis, apoptosis, or via extracellular vesicles. Animal viruses could potentially pass through the fungal cell wall and subsequently enter the fungal cell through mechanisms such as membrane fusion, endocytosis, or a nonspecific entry mechanism that may partially resemble the direct uptake of macromolecules by the fungal cells.
